# Draft Genome Sequence of “*Candidatus* Phytoplasma pruni” (X-Disease Group, Subgroup 16SrIII-B) Strain ChTDIII from Argentina

**DOI:** 10.1128/MRA.00792-20

**Published:** 2020-09-17

**Authors:** Franco Daniel Fernández, Christina Zübert, Bruno Huettel, Michael Kube, Luis Rogelio Conci

**Affiliations:** a Instituto Nacional de Tecnología Agropecuaria, Centro de Investigaciones Agropecuarias, Instituto de Patología Vegetal, Córdoba, Argentina; b Consejo Nacional de Investigaciones Científicas y Técnicas, Unidad de Fitopatología y Modelización Agrícola, Córdoba, Argentina; c University of Hohenheim, Integrative Infection Biology Crops-Livestock, Stuttgart, Germany; d Max Planck Genome Centre Cologne, Max Planck Institute for Plant Breeding, Cologne, Germany; University of Maryland School of Medicine

## Abstract

Herein, we report the draft genome sequence of “*Candidatus* Phytoplasma pruni” strain ChTDIII (subgroup 16SrIII-B). The final assembly consists of 790,517 nucleotides organized in 67 contigs (minimal size, 1 kb), with a G+C content of 29.4% and encoding 672 proteins.

## ANNOUNCEMENT

Phytoplasmas are cell wall-less bacteria that inhabit the phloem tissue of infected plants and are transmitted from plant to plant by phloem-feeding insect vectors, principally leafhoppers (1). These pathogens have been described throughout the world as affecting several hundred plant species (2). Despite numerous efforts, it has been challenging to obtain stable *in vitro* phytoplasma cultures (1), which limits the study of these pathogens. A classification scheme based on restriction fragment length polymorphism (RFLP) 16S rRNA (16Sr) sequence profiles has allowed the identification of 36 16Sr groups to date (3). The X-disease group (16SrIII) is one of the most diverse and widely distributed groups of phytoplasmas (4, 5). So far, six draft genome sequences representing the X-disease group have been described, i.e., “*Candidatus* Phytoplasma pruni” strains MA (16SrIII-B), MW1 and VAC (16SrIII-F), JR1 (16SrIII-H) (6), CX (16SrIII-A) (7), and Vc33 (16SrIII-J) (8). China tree decline phytoplasma (subgroup 16SrIII-B) is a “*Ca*. Phytoplasma pruni”-related strain that has been described in several South American countries (5). This subgroup has also been cited as infecting other plant species, such as peach (9), cassava (10), and sweet orange (Huanglongbing [HLB]-like symptoms) (11). Herein, we report the draft genome sequence of “*Ca*. Phytoplasma pruni” strain ChTDIII. The *Melia azedarach* L. ChTDIII strain was originally obtained from infected chinaberry trees (5) and was maintained and propagated by grafting. Total DNA was extracted from midribs and petioles using the DNeasy plant minikit (Qiagen) according to the manufacturer’s instructions. DNA quality was evaluated with a TapeStation with genomic tape (Agilent, Santa Clara, CA, USA), and DNA amounts were verified with a Qubit broad-range kit (Thermo Fisher Scientific, Waltham, MA, USA). Illumina-compatible libraries were generated with the NEBNext Ultra II FS DNA library preparation kit for Illumina (New England Biolabs, Ipswich, MA, USA) and sequenced via the sequencing-by-synthesis mode on a HiSeq 3000 system (Illumina, San Diego, CA, USA) in 2 × 150-bp paired-end read mode. A total of 4,970,674 paired reads were generated from the ChTDIII metagenomic DNA sample. Read trimming and *de novo* assembly were performed in CLC Genomics Workbench v8.0 (Qiagen, Aarhus, Denmark). Contigs were compared via BLASTX against the NCBI nonredundant protein database, enabling taxonomic binning in MEGAN (12). Default parameters were used except where otherwise noted. Contigs assigned to the *Mollicutes* class were used as a database for the selection of *Mollicutes*-assigned reads. This read set (316,748 reads) resulted in improved assembly of a 791-kb phytoplasma-derived draft (54-fold sequencing coverage). The draft genome assembly consists of 67 contigs with a G+C content of 29.4%, an *N*_50_ value of 31,273 nucleotides, and completeness of ∼97% according to CheckM v1.0.18 (13). Based on the NCBI Prokaryotic Genome Annotation Pipeline (PGAP) (14), 2 rRNA genes, 30 tRNA genes, and 672 protein-coding genes were annotated. The prediction of effector protein homologues was based on a previously described pipeline (15). No homologous genes for the SAP11, SAP54, and TENGU proteins were predicted; however, other proteins with a putative signal peptide domain, which could be considered as potentially homologous to the previously described SAPs (16), were identified. Interestingly, a gene for a sucrose phosphorylase homologue was found, showing sequence conservation along with orthologous properties, as described for “*Candidatus* Phytoplasma australiense,” “*Candidatus* Phytoplasma ziziphi,” and “*Candidatus* Phytoplasma asteris” strain OY-M.

The report of the draft genome for the 16SrIII-B subgroup contributes to a better understanding of the diversity and pathogenic mechanisms in the X-disease group.

### Data availability.

This whole-genome shotgun project has been deposited in DDBJ/ENA/GenBank under accession number JABUOH000000000. The version described in this paper is the first version, JABUOH010000000. Raw sequencing data have been deposited in the NCBI Sequence Read Archive (SRA) under accession number PRJNA636643.
